# NADPH Oxidase 2 Regulates NLRP3 Inflammasome Activation in the Brain after Traumatic Brain Injury

**DOI:** 10.1155/2017/6057609

**Published:** 2017-07-12

**Authors:** Merry W. Ma, Jing Wang, Krishnan M. Dhandapani, Darrell W. Brann

**Affiliations:** ^1^Charlie Norwood VA Medical Center, One Freedom Way, Augusta, GA 30904, USA; ^2^Department of Neuroscience and Regenerative Medicine, Medical College of Georgia, Augusta University, Augusta, GA, USA; ^3^Department of Neurosurgery, Medical College of Georgia, Augusta University, Augusta, GA, USA

## Abstract

Traumatic brain injury (TBI) is a leading cause of death and disability worldwide. After the initial primary mechanical injury, a complex secondary injury cascade involving oxidative stress and neuroinflammation follows, which may exacerbate the injury and complicate the healing process. NADPH oxidase 2 (NOX2) is a major contributor to oxidative stress in TBI pathology, and inhibition of NOX2 is neuroprotective. The NLRP3 inflammasome can become activated in response to oxidative stress, but little is known about the role of NOX2 in regulating NLRP3 inflammasome activation following TBI. In this study, we utilized NOX2 knockout mice to study the role of NOX2 in mediating NLRP3 inflammasome expression and activation following a controlled cortical impact. Expression of NLRP3 inflammasome components NLRP3 and apoptosis-associated speck-like protein containing a CARD (ASC), as well as its downstream products cleaved caspase-1 and interleukin-1*β* (IL-1*β*), was robustly increased in the injured cerebral cortex following TBI. Deletion of NOX2 attenuated the expression, assembly, and activity of the NLRP3 inflammasome via a mechanism that was associated with TXNIP, a sensor of oxidative stress. The results support the notion that NOX2-dependent inflammasome activation contributes to TBI pathology.

## 1. Introduction

Traumatic brain injury (TBI) is a major cause of disability in young adults and contributes to over 30% of injury-related deaths [1]. In addition to the serious financial burden on the families and society, TBI can lead to grave long-term impairments for the survivors [2, 3]. TBI is a highly complex disorder that involves a primary injury resulting in neuronal death and a secondary injury cascade involving, but not limited to, edema, excitotoxicity, mitochondrial dysfunction, oxidative stress, and inflammation [4–7]. This secondary injury can extend past the initially damaged tissue and lead to further neurological deterioration for months after the primary injury [8]. Due to the heterogeneity of TBI patients and the complex nature of secondary injury cascades following TBI, translation of neuroprotective strategies or pharmacological treatments to the clinic has proven to be a challenge.

Oxidative stress is one of the major mediators of the secondary injury following TBI. Many sources may contribute toward the cellular production of reactive oxygen species (ROS); however, NADPH oxidases (NOX) are the only family of enzymes solely devoted to the production of ROS whereas other enzymes, such as xanthine oxidase, lipooxygenase, cyclooxygenase, nitric oxide synthase, and cyp450, generate ROS as a byproduct [9]. NOX has essential physiological functions for many cellular signaling pathways and immune defense [10]. However, sustained activation, such as that involved in chronically activated microglia after TBI, is detrimental to recovery and exacerbates the primary injury [11]. Several NOX isoforms have been studied in the context of TBI pathology both in humans and in rodents [12]. Postmortem analysis demonstrated a correlation between elevated NOX2 and NOX4 expression and clinical TBI severity [13, 14], and circulating neutrophils of TBI patients show increased NOX2 expression [15]. In rodents, our laboratory showed acutely increased NOX2 expression in the cortex and CA1 hippocampus in the days following TBI [16]. Other studies reported chronically elevated NOX2 expression following TBI [11, 17], and a recent study also showed elevated NOX4 expression after TBI [14]. Inhibition of NOX2 has been shown to be neuroprotective by reducing lesion severity, apoptosis, oxidative damage, and inflammation [12, 16, 18].

Neuroinflammation is associated with the progression of neurodegenerative disorders and contributes to the secondary injury after TBI [19–22]. Inflammasomes, such as NOD-like receptors (NLRP) and absent in melanoma 2- (AIM 2-) like receptors, are innate immune system sensors of damage-associated molecular patterns (DAMPs) and pathogen-associated molecular patterns (PAMPs) that regulate the activation of caspase-1 and promote secretion of proinflammatory cytokines, such as IL-1*β* and IL-18 [23–25]. NLRP3 is the most abundant and most studied inflammasome in brain injury [26–28]. Upon sensing stimuli, NLRP3 nucleotide-binding domain (NBD) oligomerizes the pyrin domain (PYD), which serves to nucleate apoptosis-associated speck-like protein containing a CARD (ASC) proteins through PYD-PYD interactions. Long ASC filaments then form and caspase activation recruitment domain (CARD) interactions recruit pro-caspase-1 to this multimeric protein complex. The proximity of pro-caspase-1 to one another induces an autoproteolytic cleavage that activates caspase-1, leading to further release of proinflammatory cytokines [24]. Although not extensively studied, there is growing evidence that inflammasomes play a role in TBI pathology. In support of this contention, high NLRP1, ASC, AIM 2, and caspase-1 expression was detected in the CSF of TBI patients [29–31] and correlated with severity of TBI [30]. Recent studies have reported elevated NLRP3 inflammasome expression in rat [32] and in human brains after TBI [33]. Furthermore, ATP and other ROS-induced DAMPs that are released after TBI [34] can activate the NLRP3 inflammasome, suggesting a potential therapeutic role for NLRP3 after TBI.

Mitochondrial ROS reportedly can activate NLRP3 inflammasomes [35]. In particular, NOX isoforms may serve as a source of ROS for inflammasome activation, as p22phox knockdown, apocynin, and DPI all independently diminished IL-1*β* secretion [36]. ROS induction of NLRP3 activation would suggest potential involvement of redox-sensing proteins in the mechanism of ROS regulation of the inflammasome. In support of this possibility, thioredoxin-interacting protein (TXNIP) can directly activate NLRP3 inflammasome via dissociation of TXNIP from thioredoxin and subsequent binding to NLRP3 [37]. However, this has not yet been examined in the context of TBI.

The above studies suggest that NOX2 can potentially regulate NLRP3 inflammasome activation; however, several important questions remain unanswered. What is the temporal expression of the NLRP3 inflammasome in the mouse brain after TBI? Does NOX2 regulate NLRP3 inflammasome expression and complex formation, as well as downstream proinflammatory cytokines? If so, what is the mechanism mediating NOX2-derived ROS crosstalk with NLRP3 inflammasome-mediated neuroinflammation? To address these key questions, we used a NOX2 knockout (KO) mouse model to examine whether NOX2 is an essential regulator of NLRP3 inflammasome activation in TBI. The results of the study reveal that NLRP3 expression, complex formation, and activation are robustly increased in the injured mouse cerebral cortex after TBI—an effect paralleled by increased cleavage of caspase-1 with associated IL-1*β* activation. Furthermore, NOX2 appears critical for TBI-induced NLRP3 inflammasome pathway activation, as NOX2 deletion strongly attenuates the expression, complex formation, and activation of NLRP3, as well as cleavage of caspase-1 and IL-1*β* activation after TBI. Finally, the results also provide evidence that TXNIP may be a key factor mediating the crosstalk between oxidative stress and neuroinflammation.

## 2. Materials and Methods

### 2.1. Animals

Adult 3-month-old C5BL/6N male mice were obtained from Envigo (Prattville, AL) for use in this study. NOX2 KO (B6.129S-*Cybbtm1Din*/J; Stock number 002365) and WT (000664) mice of equivalent age and weight were obtained from Jackson Labs (Bar Harbor, ME). Mice were housed under humidity- and temperature-controlled conditions with free access to food and water. All animal experiments were approved by the Charlie Norwood VA Medical Center Institutional Animal Care and Use Committee.

### 2.2. Controlled Cortical Impact

Mice were anesthetized with isoflurane (2–4%) and subjected to a sham injury or controlled cortical impact as detailed previously by our laboratory [16, 38, 39]. Mice were placed in a stereotaxic frame (Leica Impact One™ Stereotaxic Impactor for CCI, Buffalo Grove, IL, USA), and a 3.5 mm craniotomy was made in the right parietal bone midway between the lambda and the bregma with the medial edge 1 mm lateral from the midline. The dura was left intact. TBI mice, but not shams, were impacted at 4.5 m/s impactor with 20 ms dwell time and 1 mm depression using a 3 mm diameter convex tip to produce a moderate TBI. Bone wax was used to cover the cranial window, and the scalp incision was closed with surgical staples. Mice were allowed to recover before being placed back in to their housing environment. Throughout the procedure, body temperature was monitored and maintained at 37°C using a small thermometer (Kopf Instruments, Tujunga, CA, USA). In experiments that utilized the NADPH oxidase inhibitor, apocynin (Sigma-Aldrich, 5 mg/kg) or saline was administered by intraperitoneal (IP) injections beginning at 23 hours after TBI and administered every 24 hours until the time of sacrifice. Sham-operated mice received identical treatment except for the cortical impact. Control animals did not undergo any procedures. Fewer mice were utilized for the sham and control groups due to their low variability within each group. WT TBI, KO TBI, and APO TBI groups indicate wild-type, NOX2 knockout, or apocynin-treated mice sacrificed at time points indicated in the text.

### 2.3. Tissue Collection

**Figure 1:**
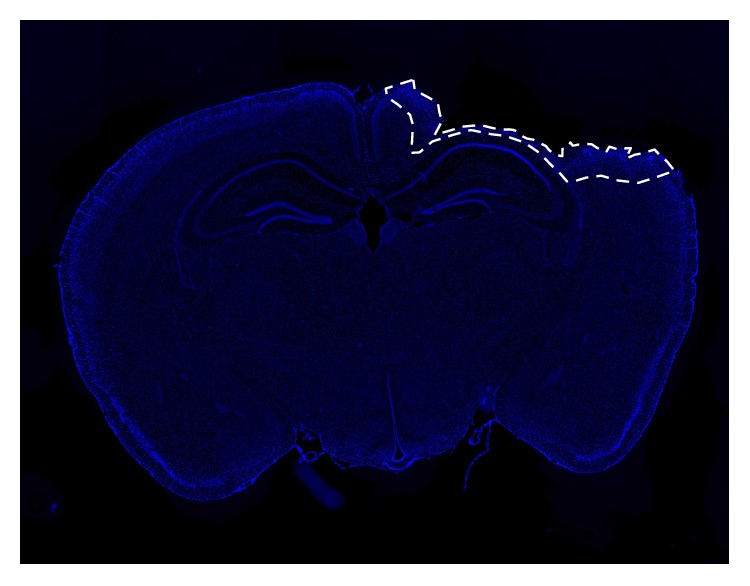
Demarcated perilesional area. Representative DAPI-stained confocal image of whole-brain slice showing perilesional area used for WB, IHC, and PCR analyses. Whole-brain image was created by stitching together 20 images taken at 4x of WT mouse at 4 d post-TBI. White-dotted outline demarcates perilesional area from where all confocal images have been taken.

All animals were transcardially perfused with ice-cold saline and decapitated at the desired time point after TBI. For RT-PCR and Western blot analysis, the brains were dissected and processed, as described in subsequent sections. For coronal sections, mice were transcardially perfused with saline and then 4% paraformaldehyde before decapitation. The perfused brains were removed, fixed in 4% paraformaldehyde for 24 hours, then cryoprotected in 30% sucrose, and sectioned on a cryostat to obtain 20 *μ*M coronal sections for further staining, immunohistochemistry, or proximity ligation assay.  Figure 1 outlines the perilesional area from where all confocal images were taken.

### 2.4. BV2 Cell Experiments

BV2, immortalized murine microglia, cells were cultured in RPMI 1640 media supplemented with 10% heat-inactivated FBS at 37°C in a humidified incubator with a 5% CO_2_ atmosphere. Samples for inflammasome-positive control were collected after cells were treated with 500 ng/mL LPS (Sigma-Aldrich, L4130) for 3 hours. 20 *μ*g protein was applied to each lane for Western blot analysis.

### 2.5. Quantification of Lesion Volume and NeuN

To quantify cortical tissue loss following CCI, coronal sections taken from the middle of the brain lesion showing the largest damage were stained with cresyl violet and imaged using a digital camera integrated with a light microscope. Using a similar method as previously described [40, 41], we took coronal sections from the center of the lesion for each mouse and assessed the area of the ipsilateral and contralateral cortices using NIH ImageJ software. The cortical lesion size was expressed as a percentage, calculated as follows: (Ac−Ai)/(Ac) × 100, where Ac is the contralateral cortical area and Ai is the ipsilateral cortical area. This method allows the quantification of lesion size as a percentage of the contralateral (uninjured) cortex of the same coronal section for each mouse. NeuN^+^ cells were counted using a method similar to that previously reported [42]. %NeuN^+^ cells were calculated as (number of NeuN^+^ cells)/(number of DAPI^+^ cells) in a consistent and set area using 40x confocal images as described below. The percentages of %NeuN^+^ cells were reported relative to that of sham mice. Sections from 4 mice (4 sections per mouse) per experimental group were examined for the lesion analysis and for quantification of neuronal survival.

### 2.6. RT-PCR

Injured cortical tissue from the perilesional area (or an anatomically matched cortical area on sham/control mice) averaging 50–60 mg per mouse was collected at various time points after TBI. RNA was isolated using the SV total RNA isolation system (Promega). Superscript III one-step RT-PCR system with platinum Taq DNA Polymerase (Invitrogen) was used for reverse transcriptase-PCR. Primers are as listed in Table 1 (Integrated DNA Technologies). Gene expression analyses were done using the comparative ΔΔCt method, and mRNA changes were expressed as fold change as compared to control animals. 18S was used as the housekeeping gene for normalization. Group average ΔCt values for NLRP3, ASC, caspase-1, and IL-1*β* are shown in Table 2.

### 2.7. Western Blot Analysis

50–60 mg of injured cortical tissue from the perilesional area (or a similar cortical area on sham/control mice) was collected at various time points after TBI as previously described by our laboratory [16, 43]. The tissue was immediately frozen in dry ice or kept on ice for immediate homogenization using a tissue tear or with ice-cold RIPA buffer. The homogenate was centrifuged at 12,000 RPM for 20 minutes at 4°C, and the supernatant was aliquoted for further analysis. Protein concentrations were determined by the BCA Protein Assay (Thermo Fisher Scientific, Carlsbad, CA). 40 *μ*g samples of protein was separated on a 4–20% SDS-PAGE gel and transferred onto 0.2 *μ*M nitrocellulose membranes. Blots were blocked with 5% bovine serum albumin for 1 hour at room temperature with gentle shaking. After blocking, the blots were incubated overnight in 4°C with the following antibodies: NLRP3 (1 : 1000, Adipogen, AG-20B-0014), ASC (1 : 200, Santa Cruz, sc-22,514-R), cleaved caspase-1 p20 (1 : 1000, Adipogen, AG-20B-0042), and IL-1*β* (1 : 1000, Abcam, ab9722). *β*-actin (1 : 4000, Sigma-Aldrich, A5441) was used as a loading control. The membrane was then washed with 1x TBST and then incubated with the secondary antibodies. Bound proteins were visualized using the Odyssey Imaging System (LI-COR Bioscience, Lincoln, NB) and analyzed with NIH ImageJ analysis software. The immunoblot densities were corrected based on corresponding *β*-actin-loading controls.

### 2.8. Confocal Microscopy and Image Analysis

Three to four coronal sections from each mouse were washed with PBS and permeabilized with 0.4% Triton-X PBS for 20 minutes. The sections were then blocked with 10% normal donkey serum for 1 hour at room temperature in a buffer containing 0.1% Triton. Sections were incubated for 2 nights with the primary antibody at 4°C in the same buffer using the following antibodies: NeuN (1 : 200, Millipore MAB377), ASC (1 : 50, Santa Cruz, sc-22,514-R), NLRP3 (1 : 50, Santa Cruz, sc-66,846), cleaved caspase-1 p20 (1 : 50, Santa Cruz, sc-22,165), cleaved IL-1*β* (1 : 50, Santa Cruz, sc-23,459), and TXNIP (1 : 50, Santa Cruz, sc-33,099). After the primary antibody incubation, the sections were washed in PBS and incubated with the appropriate secondary antibodies (1 : 500, Alexa Fluor 488/568) for 1 hour at room temperature. Sections were then mounted with water-based DAPI-mounting medium containing antifading agents and observed using confocal microscopy. All images were captured on a confocal laser microscope (Carl Zeiss, Germany) using Zen software at 40x magnification. The intensity above threshold of the fluorescent signal of the bound antibodies was analyzed using NIH ImageJ software. Data were expressed as fold change from sham.

### 2.9. Proximity Ligation (Duolink) Assay

The proximity ligation (Duolink) assay was performed, as described by our laboratory [44]. Briefly, coronal brain sections were blocked in 5% (vol/vol) donkey serum for 1 hour at room temperature and incubated overnight with the following pairs of primary antibodies: goat-NLRP3 (Santa Cruz, sc-34,408) and rabbit-ASC (Santa Cruz, sc-22,514-R); or rabbit-NLRP3 (Santa Cruz, sc-66,846) and goat-TXNIP (Santa Cruz, sc-33,099) at 4°C. These sections were then incubated for 1 hour at 37°C with the following Duolink PLA probes: anti-Rabbit MINUS (Sigma-Aldrich, DUO92005) and anti-goat PLUS (Sigma-Aldrich, DUO92003). Duolink in situ detection reagent kit (Sigma-Aldrich, DUO92008) was used for ligation and amplification at 37°C using the according to the manufacturer's protocol. All sections were then mounted on a slide using DAPI-mounting media, and all images were captured on a confocal laser microscope (Carl Zeiss, Germany) using the Zen software at 40x magnification. Fluorescence of PLA indicating interacting proteins was analyzed as intensity above threshold using NIH ImageJ software and represented as fold change from shams.

### 2.10. Statistical Analysis

An independent two-sample *t*-test was conducted to investigate the difference between lesion volume of WT versus KO TBI mice. The one-way ANOVA test was conducted to analyze the differences within control, sham, and the different time points following TBI. The one-way ANOVA test was also used to investigate whether there is a significant difference among control, sham, WT, KO, and APO (apocynin) mice for all proteins in the study. Whenever an ANOVA test was found to be significant, the post hoc Tukey's test was conducted to make pairwise comparisons between the groups of animals. Statistical significance was accepted at the 95% confidence level (*P* < 0.05) using GraphPad Prism. Data was expressed as mean ± standard error (SEM).

## 3. Results

### 3.1. Mice Deficient in NOX2 Have Reduced Lesion Size and Neuronal Cell Death after TBI

**Figure 2:**
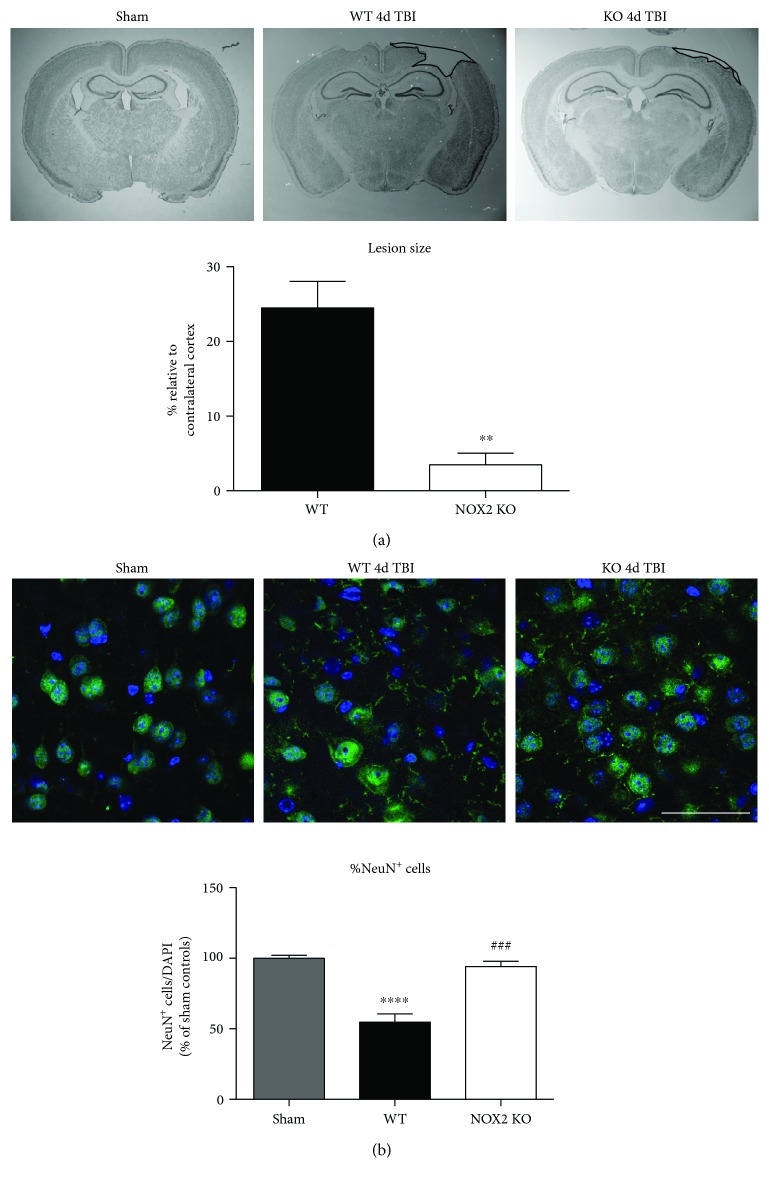
Mice deficient in NOX2 have both reduced lesion size and reduced neuronal damage after TBI. (a) Representative cresyl violet staining on day 4 after TBI from sham, WT TBI, and NOX2 KO TBI mice show neurons. Images have been converted to grayscale for added clarity. Mice were sacrificed at 4 days after TBI, and the brains of sham, WT TBI, and NOX2 KO TBI were collected for sectioning into 20 *μ*M slices. Lesion size of TBI mice was calculated as a percent relative to a similarly affected area from the contralateral cortex of the same section. Lesion areas used for quantification are outlined in black. NOX2 KO mice show reduced lesion volume on gross examination throughout the injured cortex as quantified to the right. *n* = 4–6 mice/group. (^∗∗^*p* < 0.01 WT versus NOX2 KO) (b) Representative confocal images showing NeuN (green) and DAPI (blue) fluorescent signal of the injured cortex in sham, WT, and NOX2 KO mice at the 4-day post-TBI. NeuN/DAPI double-positive cells were counted and analyzed as a percent of total DAPI^+^ cells, which is shown below the representative panel. TBI reduces NeuN-positive cells in the injured cortex, which is attenuated with deletion of NOX2. *n* = 4 mice/group. Scale bar represents 50 *μ*M. (^∗∗∗∗^*p* < 0.0001 sham versus WT; ^###^*p* < 0.001 WT versus KO).

Figure 2(a) shows representative cresyl violet staining of brain sections from sham, WT, and NOX2 KO mice to examine lesion size. As shown in Figure 2(a), sham-injured mice show no gross lesion or noticeable damage to the cerebral cortex. In contrast, WT animals undergoing CCI exhibit a moderately sized lesion, which is significantly reduced in NOX2 KO mice. To further quantitate the findings, lesion volume was calculated at the center of the lesion on the largest injured area (outlined in black) and compared to anatomically matched cortical sections. As shown in Figure 2(a), NOX2 KO mice have a significant reduction in lesion size, as compared to WT mice. Closer examination of the injured cortex under confocal microscopy revealed that TBI decreases the number of NeuN^+^ neurons in the injured cortex in WT, whereas mice deficient in NOX2 retain similar neuronal densities as sham mice (Figure 2(b)). These findings indicate that the deletion of NOX2 offers robust neuroprotection after TBI.

### 3.2. TBI Induces NLRP3 Inflammasome Expression in the Cortex

**Figure 3:**
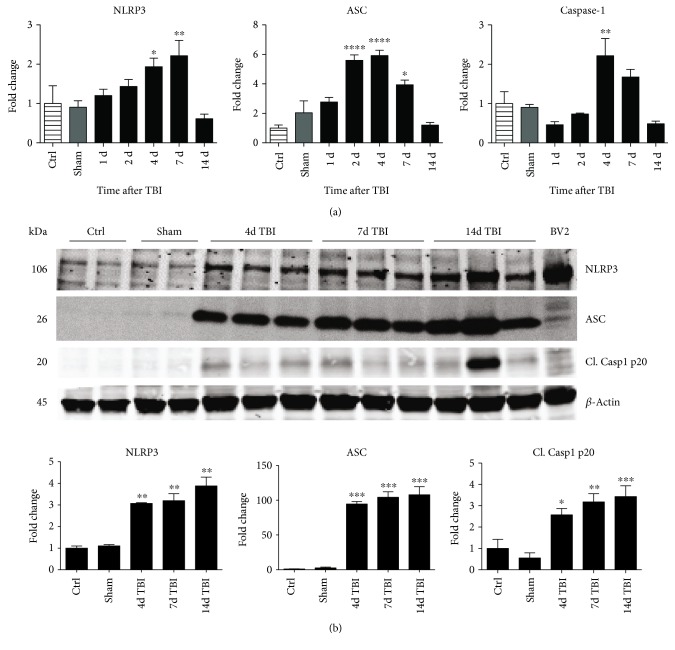
Time course gene and protein expression of NLRP3 inflammasome components in the mouse cerebral cortex after TBI. (a) Temporal pattern of NLRP3, ASC, and caspase-1 gene expression after TBI in WT mice between 1- and 14-day post-TBI showing peak increased mRNA expression between 4 and 7 days after TBI. *n* = 4, 7, 4, 7, 6, 13, and 4 mice (ctrl, sham, 1d, 2d, 4d, 7d, and 14d, resp.). (b) Representative Western blots showing temporal protein expression of NLRP3, ASC, and cleaved caspase-1 p20 after TBI in WT mice at 4, 7, and 14 days after TBI. Quantification of all blots shown below the image indicating increased protein expression of NLRP3, ASC, and cleaved caspase-1 after TBI (data normalized to *β*-actin and presented as fold change relative to ctrl mice). BV2 sample included as positive control. *n* = 4, 4, 6, 6, and 6 mice (ctrl, sham, 4d, 7d, and 14d, resp.). (^∗^*p* < 0.05, ^∗∗^*p* < 0.01, ^∗∗∗^*p* < 0.001, and ^∗∗∗∗^*p* < 0.0001 sham versus TBI).

We next hypothesized that NOX2 may play a role in NLRP3 inflammasome activation after TBI. To test this hypothesis, we first performed a time course examining expression of NLRP3 inflammasome pathway factors in the cerebral cortex after TBI. As shown in Figure 3(a), RT-PCR analysis revealed that NLRP3 mRNA levels in the cortex increased 2.6-fold at 4 days and 3-fold at 7 days after TBI in WT mice (relative to uninjured sham mice). Similarly, ASC mRNA expression was significantly increased at 2–7 days after TBI, showing a 6-fold increase at peak elevation compared to sham. Expression of pro-caspase-1, a substrate of the NLRP3 inflammasome, also was significantly elevated at 4 days after TBI showing a 2.5-fold increase. In line with the RT-PCR results, Western blot analysis revealed that NLRP3 and ASC protein levels were elevated in the cortex at 4, 7, and 14 days after TBI (Figure 3(b)). Furthermore, p20, the cleaved product of caspase-1, showed a time-dependent increase in cleaved caspase-1 expression within the cerebral cortex following TBI.

### 3.3. NOX2 Deletion or Inhibition Leads to a Significantly Reduced Induction of NLRP3 Inflammasome Pathway Factor Expression in the Injured Cortex Following TBI

**Figure 4:**
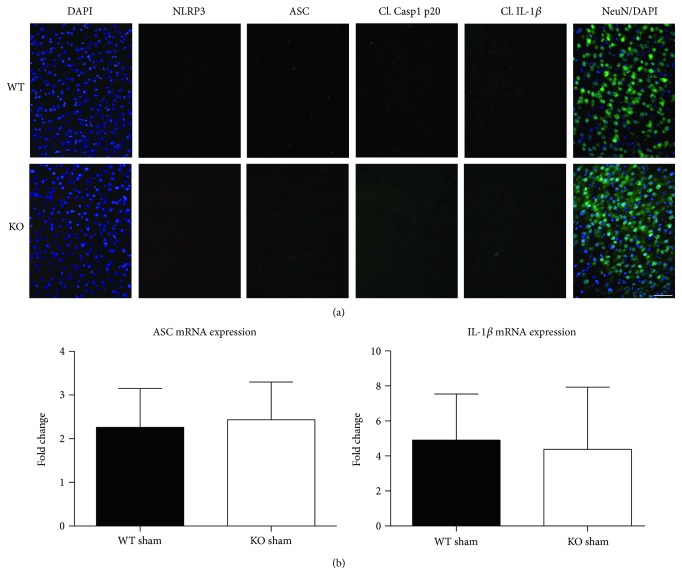
WT and KO mice show no differences in expression of NLRP3 inflammasome components at baseline. (a) Representative confocal images show baseline immunoreactivity of NLRP3, ASC, cleaved caspase-1 (p2), cleaved IL-1*β*, and NeuN in WT and KO sham mice. No differences in the expression of inflammasome components were observed between WT and KO sham mice. Scale bar represents 50 *μ*M. (b) Quantitative RT-PCR results for ASC and IL-1*β*, genes that appeared slightly elevated due to sham surgery. No differences were observed between WT and KO sham mice at the 4-day postinjury in the mRNA expression of ASC and IL-1*β*. *n* = 4, 3 mice (WT, sham, and KO sham, resp.).

**Figure 5:**
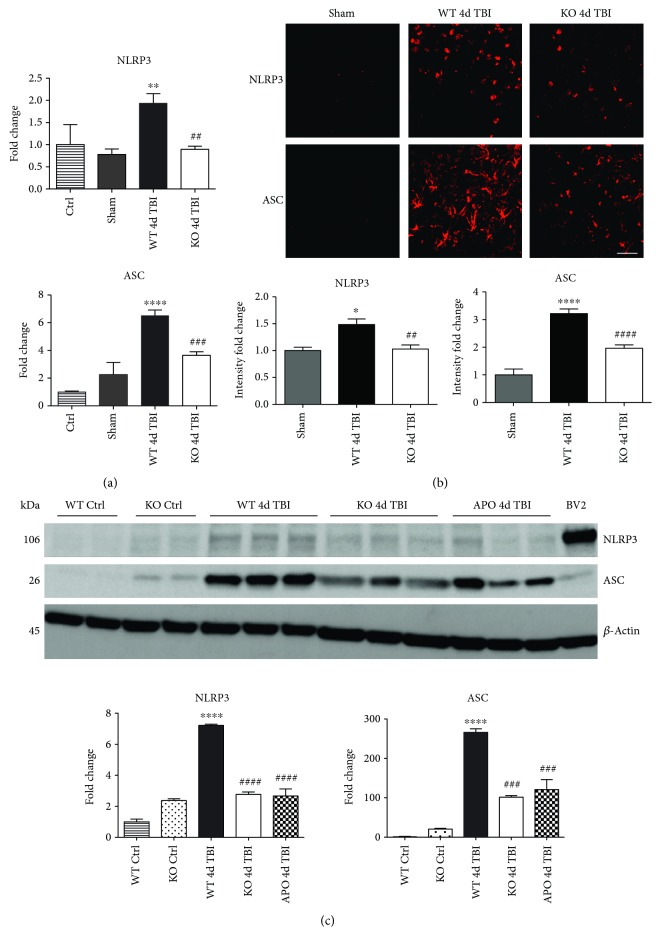
Deficiency of NOX2 reduces gene and protein expression of NLRP3 and ASC in the mouse cerebral cortex after TBI. (a) Deletion of NOX2 attenuates mRNA gene expression of NLRP3 and ASC at 4 days after TBI as compared to WT mice. *n* = 4, 6, 6, and 8 mice (ctrl, sham, WT, and KO, resp.). (b) Representative confocal images show that NLRP3 and ASC immunoreactivity is increased at the 4-day post-TBI in the WT-injured cortex. Deletion of NOX2 attenuates the expression of NLRP3 and ASC in the injured cortex following TBI. All images quantified below representative panel (data presented as fold change relative to sham mice). *n* = 4, 6, and 6 mice (sham, WT, and KO, resp.). Scale bar represents 50 *μ*M. (c) Representative Western blot and quantification of all blots show the protein expression of NLRP3 and ASC in WT, NOX2 KO, and apocynin-treated mice at the 4-day post-TBI (data normalized to *β*-actin and presented as fold change relative to WT ctrl mice). Both the deletion of NOX2 and inhibition of NOX attenuated NLRP3 and ASC protein levels. BV2 sample included as positive control. *n* = 4, 4, 7, 7, and 4 mice (WT ctrl, KO ctrl, WT, KO, and APO, resp.). (^∗^*p* < 0.05, ^∗∗^*p* < 0.01, and ^∗∗∗∗^*p* < 0.0001 sham versus WT TBI; ^##^*p* < 0.01, ^###^*p* < 0.001, and ^####^*p* < 0.0001 WT TBI versus KO or APO TBI).

**Figure 6:**
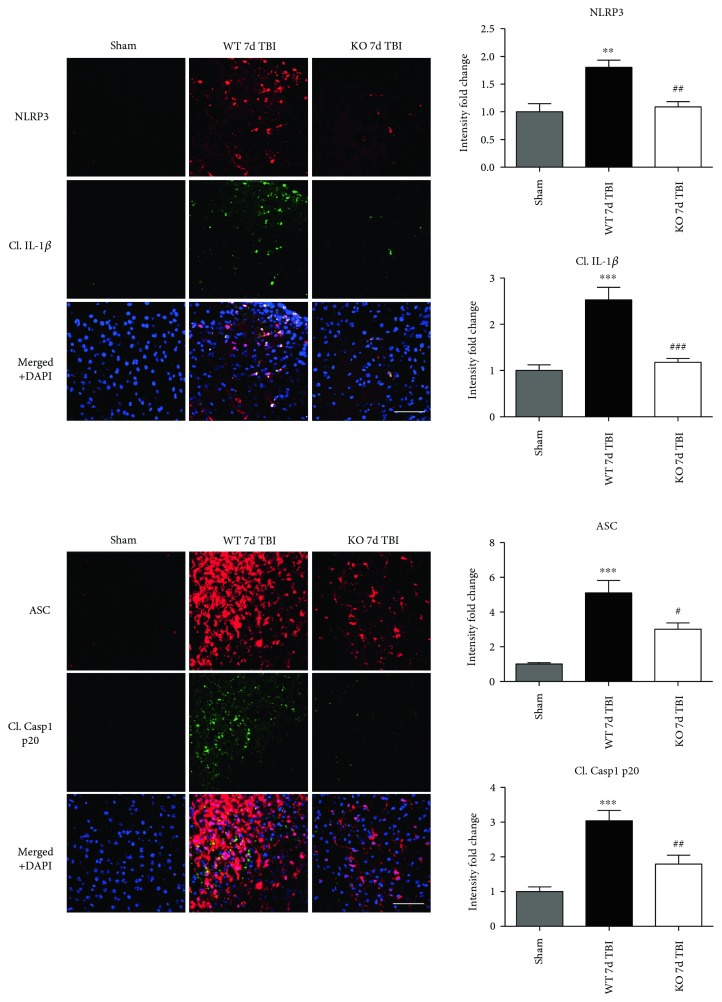
Deletion of NOX2 attenuates immunoreactivity of the injured mouse cortex to NLRP3 inflammasome and its products at the7-day post-TBI. Representative confocal images show that NLRP3, ASC, cleaved caspase-1 (p20), and cleaved IL-1*β* are elevated at the 7-day post-TBI in WT-injured mouse cortex. Deletion of NOX2 attenuates the expression of both the inflammasome and its products in the injured cortex at the 7-day post-TBI. All confocal images were quantified to the right of the representative panel of images (presented as fold change relative to shams). *n* = 4, 5, and 4 mice (sham, WT, and KO, resp.). Scale bar represents 50 *μ*M. (^∗∗^*p* < 0.01, ^∗∗∗^*p* < 0.001, sham versus WT TBI; ^#^*p* < 0.05, ^##^*p* < 0.01, and ^###^*p* < 0.001 WT TBI versus KO TBI).

**Figure 7:**
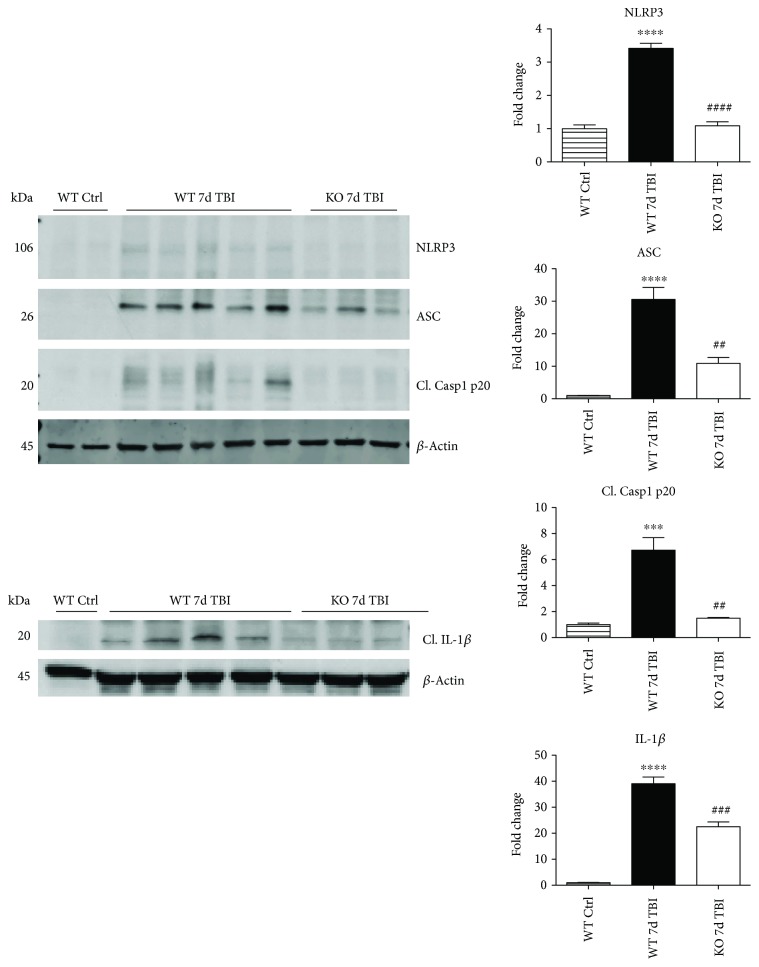
Deletion of NOX2 suppresses protein expression of NLRP3 inflammasome and its products at the 7-day post-TBI. Representative Western blots and quantification of all blots show the protein expression of NLRP3, ASC, cleaved caspase-1 (p20), and IL-1*β* in WT and NOX2 KO mice at the 7-day post-TBI. Deletion of NOX2 was able to suppress expression of the NLRP3 inflammasome and its products caspase-1 and IL-1*β* at this later time point. Values presented as fold change relative to WT ctrl mice. *n* = 4, 5, and 3 mice (sham, WT, and KO, resp.). (^∗∗∗^*p* < 0.001, ^∗∗∗∗^*p* < 0.0001 sham versus WT TBI; ^##^*p* < 0.01, ^###^*p* < 0.001, and ^####^*p* < 0.0001 WT TBI versus KO TBI).

Since ROS can stimulate NLRP3 inflammasome activation in vivo and in vitro [45], and NOX2 is a major generator of superoxide; we therefore sought to determine the role of NOX2 in induction of NLRP3 inflammasome pathway factor expression after TBI. Based on our current findings, the NLRP3 inflammasome appears to be strongly induced at the 4-day post-TBI time point. We examined the effects of NOX2 deletion on NLRP3 inflammasome components using NOX2 KO mice. We saw no significant baseline differences between WT and KO mice (Figure 4()). NOX2 KO mice exhibited a significant reduction in NLRP3 and ASC gene expression at the day-4 post-TBI, as compared to WT mice (Figure 5(a)). Furthermore, increased immunoreactivity of NLRP3 and ASC within the perilesional cortex at the 4-day post-TBI in WT mice was significantly attenuated in NOX2 KO mice (Figure 5(b)). Examination of cortical sections at the 7-day post-TBI time point showed similar attenuation of NLRP3 and ASC immunoreactivity with deletion of NOX2 (Figure 6()). To further confirm these results, we subjected cortical lysates from WT and NOX2 KO groups to Western blot analysis. We also included an additional group in which animals were treated with a NOX inhibitor, apocynin, so as to further confirm our NOX2 KO findings. Representative Western blots and densitometric analysis show that both NOX2 deletion and inhibition of NOX2 by apocynin significantly attenuated NLRP3 and ASC protein expression at 4 days after TBI, as compared to the WT group (Figure 5(c)). Similar Western blot results were obtained at 7 days after TBI where NOX2 KO mice showed attenuation of NLRP3 and ASC protein expression as compared to WT mice (Figure 7()).

### 3.4. Reduced NLRP3 Inflammasome Complex Formation in the Injured Cortex of NOX2 KO Mice Following TBI

**Figure 8:**
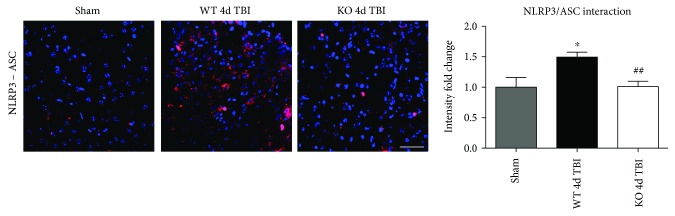
NOX2 deletion leads to a decrease in NLRP3 inflammasome complex formation in the injured mouse cortex after TBI. Proximity ligation assay (PLA) demonstrates NLRP3-ASC complex formation after TBI and regulation by NOX2. Representative confocal images of Duolink in situ co-IP show red fluorescence indicative of NLRP3-ASC protein-protein interaction in the injured cortex at the 4-day post-TBI. Deletion of NOX2 attenuates NLRP3-ASC complex formation at the 4-day post-TBI in the injured cortex. Quantification of all images shows a significant increase in NLRP3 inflammasome complex formation after TBI that is significantly attenuated by NOX2 deletion (data presented as fold change relative to sham mice). *n* = 4, 7, and 8 mice (sham, WT, and KO, resp.). Scale bar represents 50 *μ*M. (^∗^*p* < 0.05 sham versus WT TBI; ^##^*p* < 0.01 WT TBI versus KO TBI).

**Figure 9:**
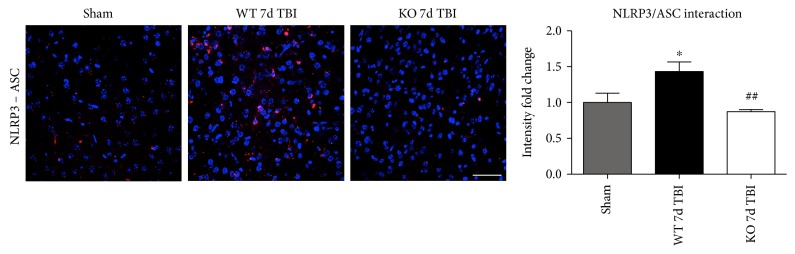
Deletion of NOX2 attenuates NLRP3 inflammasome complex formation in the injured mouse cortex at the 7-day post-TBI. Representative confocal images of Duolink in situ co-IP show red fluorescence indicative of NLRP3-ASC protein-protein interaction in the injured cortex at the 7-day post-TBI. Deletion of NOX2 attenuates the NLRP3-ASC complex formation at the 7-day post-TBI in the injured cortex. Quantification of all images shows a significant increase in NLRP3 inflammasome complex formation after TBI that is significantly attenuated by NOX2 deletion (presented as fold change relative to shams). *n* = 4, 6, and 6 mice (sham, WT, and KO, resp.). Scale bar represents 50 *μ*M. (^∗^*p* < 0.05 sham versus WT TBI; ^##^*p* < 0.01 WT TBI versus KO TBI).

Assembly of NLRP3 inflammasome components into a complex is necessary for functional activation. We therefore used an in situ Duolink coimmunoprecipitation assay to measure the protein-protein interaction of NLRP3 and ASC in the injured mouse cortex at 4 days after TBI. TBI increased NLRP3-ASC complex formation, as visualized using confocal microscopy, in the injured cerebral cortex at the 4-day post-TBI, as compared to sham controls (Figure 8). Examination at 7 days after TBI revealed similar reduction in the NLRP3 inflammasome complex formation with deletion of NOX2 (Figure 9). Notably, deletion of NOX2 significantly suppressed the TBI-induced NLRP3-ASC complex formation in the injured cortex, indicating that NOX2 regulates both NLRP3 inflammasome expression and the complex formation after TBI.

### 3.5. NOX2 Regulates Caspase-1 Expression and Activity after TBI

**Figure 10:**
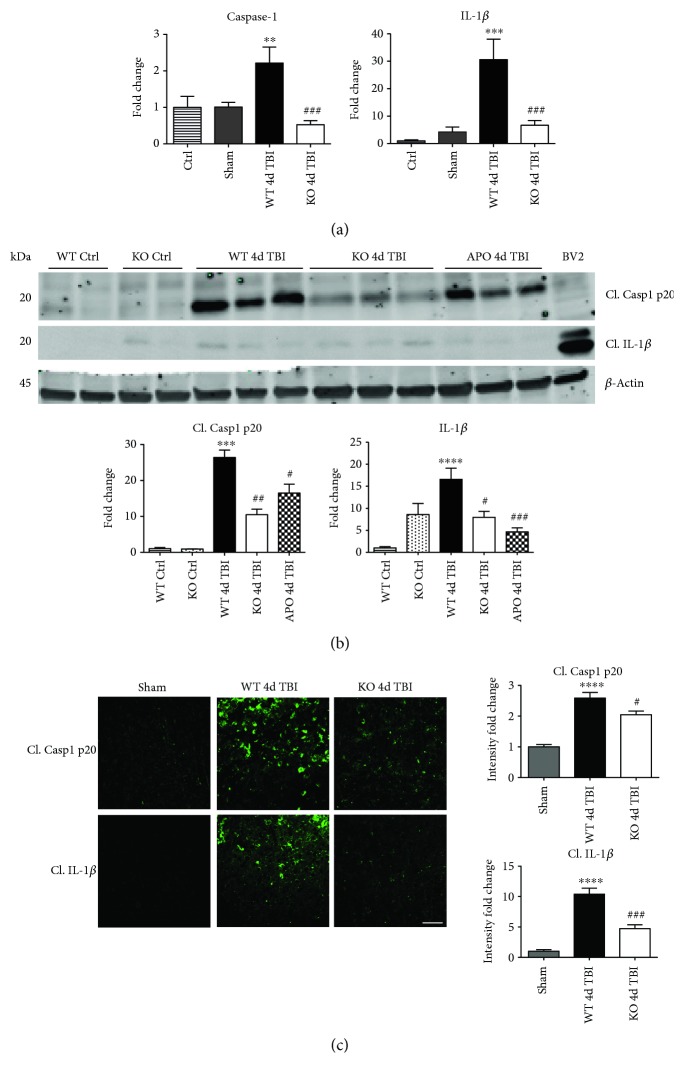
NOX2 deletion leads to a decrease in expression and activation of the NLRP3 inflammasome effectors, caspase-1 and IL-1*β* in the injured cortex after TBI. (a) Quantitative RT-PCR for inflammasome components and downstream interleukins caspase 1 and IL-1*β*. mRNA samples were collected from the injured cortex at the 4-day post-TBI in WT and NOX2 KO mice. Caspase-1 and IL-1*β* mRNA show increased gene expression at the 4-day post-TBI. Deletion of NOX2 significantly attenuates these changes. *n* = 4, 6, 6, and 9 mice (ctrl, sham, WT, and KO, resp.). (b) Representative Western blot showing protein expression of NLRP3 inflammasome products cleaved caspase-1 p20 and cleaved IL-1*β*. At 4 days after TBI, WT mice show increased expression of both cleaved effectors. However, mice deficient in NOX2 show attenuated cleavage of caspase-1 and IL-1*β*. Inhibition of NOX using apocynin also produces similar attenuated cleavage. Quantification of blots shown below representative image (data normalized to *β*-actin and presented as fold change relative to WT ctrl mice). BV2 sample included as positive control. *n* = 4, 4, 7, 7, and 4 mice (WT ctrl, KO ctrl, WT, KO, and APO, resp.). (c) Representative confocal images showing immunoreactivity of cleaved caspase 1 p20 and cleaved IL-1*β* in the injured cortex after TBI for WT and NOX2 KO mice. All images have been quantified to the right of the representative panel (data presented as fold change relative to sham mice). TBI increased cleavage of caspase-1 and IL-1*β* as detected by immunoreactivity of its cleaved products in WT mice at the 4-day post-TBI. Deletion of NOX2 attenuates this cleavage of caspase-1 and IL-1*β* at the 4-day post-TBI. *n* = 4, 6, and 6 mice (sham, WT, and KO, resp.). Scale bar represents 50 *μ*M. (^∗∗^*p* < 0.01, ^∗∗∗^*p* < 0.001, ^∗∗∗∗^*p* < 0.0001 sham versus WT TBI; ^#^*p* < 0.05, ^##^*p* < 0.01, and ^###^*p* < 0.001 WT TBI versus KO or APO TBI).

**Figure 11:**
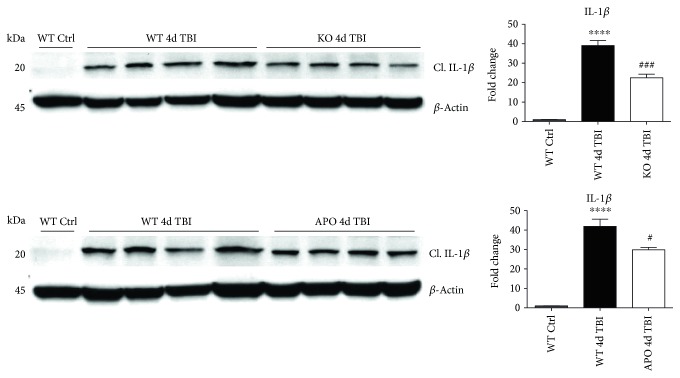
Additional Western blots for IL-1*β*. Blot showing cleaved IL-1*β* product in different set of WT ctrl, WT 4d TBI, and KO 4d TBI mice and an additional APO 4d TBI mouse from those depicted in Figure 10. Densitometry quantification represents samples in this set of blots relative to respective loading controls. Inhibition of NOX2 via either knockout or apocynin reduces protein expression of cleaved IL-1*β*. Data presented as fold change relative to WT ctrl mice. (^∗∗∗∗^*p* < 0.0001 WT ctrl versus WT TBI; ^#^*p* < 0.05 WT TBI versus APO TBI; ^###^*p* < 0.001 WT TBI versus KO TBI).

We next sought next to examine the gene expression of the NLRP3 downstream effectors, caspase-1 and IL-1*β* in the injured cerebral cortex at the 4-day post-TBI. As shown in Figure 10(a), mRNA expression of caspase-1 and IL-1*β* was increased at the 4-day post-TBI in the injured cerebral cortex of WT, but not NOX2 KO, mice. Since the activation of the inflammasome leads to increased caspase-1 cleavage, which in turn cleaves IL-1*β* into the mature form, we next utilized Western blot analysis to assess the cleavage of caspase-1 and IL-1*β* into their mature forms in the injured cortex at the 4-day post-injury. TBI significantly increased the cleavage of both caspase-1 and IL-1*β* in the injured cortex, effects that were significantly attenuated in NOX2 KO mice or by apocynin treatment (Figure 10(b)). Additional blots confirming these results for expression of cleaved IL-1*β* in WT, KO, and apocynin-treated groups are shown in Figure 11. This attenuation in cleavage is also observed at the 7-day post-TBI time point (Figure 7). The cleavage of caspase-1 and IL-1*β* is further confirmed via immunofluorescent labeling of their cleaved products (Figure 10(c)). Similarly, immunoreactivity of the cleaved caspase-1 subunit, p20, and of cleaved IL-1*β* was reduced within the perilesional cortex of NOX2 KO mice after TBI (Figure 10(c)). The examination of the 7-day post-TBI sections also showed similar attenuation of caspase-1 and IL-1*β* cleavage (Figure 6).

### 3.6. TXNIP Links NOX2-Dependent Oxidative Stress and NLRP3 Inflammasome Activation

**Figure 12:**
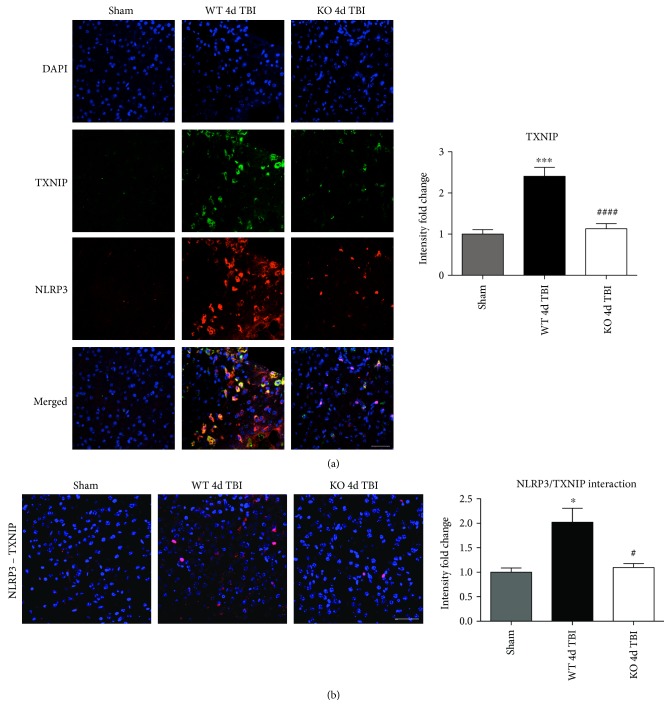
NOX2 deletion significantly attenuates TBI-induced TXNIP expression and complex formation within the injured mouse cortex. (a) Expression of TXNIP in the injured cortex after TBI. Representative images from sham, WT, and NOX2 KO mice show that TBI increases expression of TXNIP in the injured cortex after TBI, and TXNIP colocalizes with NLRP3 expression. Deletion of NOX2 attenuates the expression of TXNIP in the injured cortex at the 4-day post-TBI. Immunoreactivity from all images has been quantified to the right of the representative panel (data presented as fold change relative to shams). *n* = 4, 6, and 6 mice (sham, WT, and KO, resp.). (b) In situ PLA demonstrating NLRP3-TXNIP complex formation after TBI and regulation by NOX2. Representative confocal images of Duolink in situ co-IP show red fluorescence indicative of NLRP3-TXNIP protein-protein interaction in the injured cortex at the 4-day post-TBI. Mice deficient in NOX2 show reduced NLRP3-TXNIP complex formation at the 4-day post-TBI in the injured cortex. Quantification of all Duolink images shows significantly increased NLRP3-TXNIP interaction after TBI that is attenuated by NOX2 deletion. *n* = 4, 6, and 6 mice (sham, WT, and KO, resp.). Scale bar represents 50 *μ*M. Data for (a) and (b) presented as fold change relative to sham mice. (^∗^*p* < 0.05, ^∗∗∗^*p* < 0.001 sham versus WT TBI; ^#^*p* < 0.05, ^####^*p* < 0.0001 WT TBI versus KO TBI).

**Figure 13:**
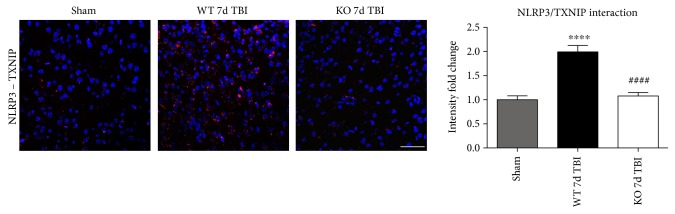
Deletion of NOX2 attenuates NLRP3-TXNIP complex formation at the 7-day post-TBI in the injured mouse cortex. Representative confocal images of Duolink in situ co-IP show red fluorescence indicative of NLRP3-TXNIP complex formation in the injured cortex at the 7-day post-TBI. Mice deficient in NOX2 show reduced NLRP3-TXNIP interaction at the 7-day post-TBI in the injured cortex. Quantification of all Duolink images shows significantly increased NLRP3-TXNIP interaction after TBI that is attenuated by NOX2 deletion (presented as fold change relative to shams). *n* = 4 mice/group. Scale bar represents 50 *μ*M. (^∗∗∗∗^*p* < 0.0001 sham versus WT TBI; ^####^*p* < 0.0001 WT TBI versus KO TBI).

TXNIP, which directly links oxidative stress to NLRP3 inflammasome formation [37], was next examined by dual immunofluorescent labeling of NLRP3 (red) and TXNIP (green). As shown in Figure 12(a), TBI significantly increased expression of TXNIP in the injured cortex of WT mice at the 4-day post-TBI, as compared to sham mice; however, this effect was significantly attenuated in NOX2 KO mice (Figure 12(a)). Of note, a NOX2-dependent colocalization of TXNIP and NLRP3 was observed after TBI. We next examined protein-protein interaction between TXNIP and NLRP3 using the Duolink proximity ligand assay. The increased interaction of TXNIP and NLRP3 after TBI was reversed in NOX KO mice at 4 days after injury (Figure 12(b)). Examination of injured brain sections at the 7-day post-TBI also showed attenuated TXNIP-NLRP3 interaction in NOX2-deficient mice (Figure 13). Thus, TXNIP may link NOX2-dependent oxidative stress and NLRP3 inflammasome activation in TBI.

## 4. Discussion

The current study provides several important findings. First, it demonstrates that NLRP3 inflammasome expression and activation are strongly induced in the injured mouse cerebral cortex after TBI. Secondly, it demonstrates that the induction of the NLRP3 inflammasome after TBI is coupled with increased interaction with TXNIP, a known activator of NLRP3. Thirdly, it provides the novel insight that NOX2 deletion strongly attenuates NLPR3 inflammasome activation after TBI, an effect that correlated with a reduced interaction of NLRP3 and TXNIP. The results of our studies were confirmed using multiple approaches, which demonstrated that NOX2 regulation of the NLRP3 inflammasome is exerted at levels of gene, protein, and complex assembly.

The complex secondary cascade of injury following TBI involves oxidative stress and inflammation. NOX2 and microglial activation are detrimental after TBI [11, 12, 16, 18, 46, 47]. Though the study of inflammasomes in the context of TBI is relatively recent, the detrimental role of elevated IL-1*β* after TBI is well documented [48–50]. Our results demonstrate that therapeutic targeting of NOX2 after TBI may attenuate this inflammatory secondary injury cascade involving IL-1*β* via a mechanism involving the NLRP3 inflammasome. We and others previously showed that NOX2 expression and NADPH oxidase activity increase rapidly in the mouse cerebral cortex and hippocampal CA1 region after TBI with a prolonged peak from 24–96 hours after TBI that occurs in microglia [12, 16, 18]. That NOX2 has a critical role in TBI outcome is evidenced by our finding that cortical lesion size is significantly decreased and neuronal survival robustly increased in NOX2 deletion mice, which agrees with previous studies by our group and others showing a similar protective effect of NOX2 deletion and NOX2 inhibitors after TBI [16, 18]. Furthermore, NOX2 inhibition has been shown to lead to improved neurological outcome after TBI [46].

We utilized a NOX2 KO mouse model of TBI to elucidate the role of NOX2 in inflammasome activation following TBI. We also utilized a selective NOX inhibitor, apocynin, to further confirm the NOX2 genetic deletion results. Both deletion and inhibition of NOX2 decreased the expression and activation of the NLRP3 inflammasome following TBI. These changes were paralleled by a concomitant reduction in IL-1*β*, supporting a regulatory role of NOX2 in proinflammatory activation after TBI. While suggestive of a role for NOX2 in NLRP3 regulation, the use of apocynin may be a caveat. Although apocynin inhibits NOX2 assembly [51–53] and requires NOX2 to elicit protection after cerebral infarction [54], apocynin also may produce anti-inflammatory and antioxidant effects independent of NOX2 [55, 56]. Despite this mechanistic limitation, apocynin exhibits documented clinical safety in asthma and chronic obstructive pulmonary disease patients [57, 58] and reduced inflammasome formation, even when administered up to 24 hours post-TBI. As 24 hours is beyond the peak expression of NOX2 in neurons [16], the delayed NOX2 elevation in immune cells may mediate release of proinflammatory cytokines, such as IL-1*β*, to exacerbate the secondary injury after TBI. Thus, our studies provide translational value supporting the therapeutic use of apocynin to reduce neuroinflammation within a delayed, clinically relevant therapeutic window after TBI.

Based on the results of our study, we propose that NOX2-derived oxidative stress induces TXNIP interaction with NLRP3 to lead to NLRP3 inflammasome activation, which exacerbates inflammation in the injured cortex after TBI. It is possible that NOX2 may indirectly regulate inflammasome-mediated neuroinflammation via altering migratory behavior and/or inflammatory phenotype of peripheral immune cells that infiltrate the injured cortex [59–61]. However, Kumar et al. reported previously that CD45^hi^ cell numbers did not vary between WT and NOX2^−/−^ mice when examining the injured cortex at the 3-day post-TBI, suggesting that NOX2 did not affect the numbers of infiltrating peripheral macrophages [60]. The same study also determined NOX2 involvement in microglial polarization following TBI [60], but whether polarization differences contribute to NLRP3 activation is unknown. Since both resident and peripheral immune cells are implicated in neuroinflammation after TBI, further studies are needed to address the involvement of inflammasomes in the infiltrating macrophages that migrate to the injured cortex following TBI.

**Figure 14:**
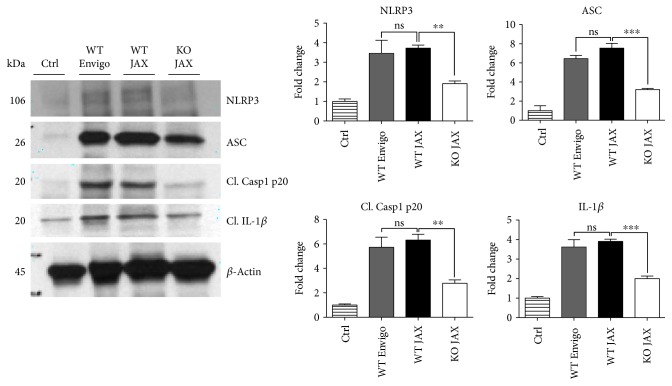
Effect of NOX2 deletion on NLRP3 inflammasome expression is conserved in both littermate and nonlittermate C57BL/6 mice at the 4-day post-TBI. Representative Western blots showing expression of NLRP3, ASC, cleaved caspase-1 (p20), and cleaved IL-1*β* in ctrl, WT TBI mice from Envigo (nonlittermate) and Jackson Labs (JAX, littermate to KO mice), and NOX2 KO TBI mice from JAX at the 4-day post-TBI. Densitometry quantification (normalized to *β*-actin and represented as fold change to ctrl) is shown to the right of the images. No differences were observed between WT mice from Envigo and JAX after TBI. Deletion of NOX2 still reduced expression of NLRP3 inflammasome components when compared to WT Jackson Labs mice at the 4-day post-TBI. *n* = 3, 3, 5, and 3 (ctrl, WT Envigo, WT JAX, KO JAX, resp.). (^∗∗^*p* < 0.01, ^∗∗∗^*p* < 0.001, ns = not significant).

While our findings suggest that NOX2 mediates NLRP3 inflammasome activation following TBI, we cannot rule out the possibility that other NOX isoforms may also contribute to inflammasome activation, as NOX4 is elevated in both rodent and human cortex after TBI [14]. Furthermore, NOX4 has been reported to regulate NLRP3 inflammasome activation in human umbilical vein endothelial cells under high-glucose environment and to modulate TXNIP [62]. Although NOX2 did not regulate NLRP3 or TXNIP in the umbilical vein endothelial cells [62], NOX2 deletion attenuated NLRP3 induction in the cerebral cortex of mice after ischemic stroke [63], as we observed after TBI. The reason for the divergent effects is not clear, but it could suggest that NOX isoform regulation of NLRP3 inflammasome activation may be tissue- and/or context specific. Importantly, these observed effects were independent of strain and supplier, further supporting the notion that NOX2 inhibition on NLRP3 inflammasome is a conserved mechanism of injury after TBI (Figure 14), and this relationship may apply to other disease models utilizing different mouse strains.

An interesting question is whether NOX2 regulates other types of inflammasomes in addition to the NLRP3 inflammasome. While our study did not address this issue, correlational human studies suggested involvement of NLRP1 and AIM 2 in the pathogenesis of TBI [29–31]; however, these findings remain to be demonstrated in mechanistic experimental models. In addition, while there is significant evidence that ROS can regulate the NLRP3 inflammasome, there is little evidence of similar ROS regulation of NLRP1 and AIM 2. Thus, further work is needed to elucidate the role of other inflammasome complexes in TBI and determine any potential regulation by NOX.

In conclusion, the results of our study demonstrate that increased NLRP3 inflammasome activation is in the injured cortex after TBI. Notably, we show that NOX2 regulates NLRP3 inflammasome expression and activation. Furthermore, we show that NOX2 regulation of the NLRP3 inflammasome may be through oxidative stress sensing of TXNIP. These findings provide new insight into the anti-inflammatory effects of NOX2 inhibition and support the potential translational value for NOX inhibitors in the clinical management of TBI. Thus, therapeutic targeting of NLRP3 inflammasome may provide a novel and efficacious treatment for TBI, as well as other acute and chronic brain injuries involving the activation of the NLRP3 inflammasome.

## Figures and Tables

**Table 1:** List of RNA primers used for RT-PCR.

Gene	Forward	Reverse
18S	5′ GTAACCCGTTGAACCCCATT 3′	5′ CCATCCAATCGGTAGTAGCG 3′
NLRP3	5′ GTTCTGAGCTCCAACCATTCT ′3	5′ CACTGTGGGTCCTTCATCTTT 3′
ASC	5′ CAGAGTACAGCCAGAACAGGACAC 3′	5′ GTGGTCTCTGCACGAACTGCCTG 3′
Caspase-1	5′ GGGCAAAGAGGAAGCAATTTATC 3′	5′ GTGCCTTGTCCATAGCAGTAA 3′
IL-1*β*	5′ AGAGCATCCAGCTTCAAATCTC 3′	5′ CAGTTGTCTAATGGGAACGTCA 3′

**Table 2:** Average ΔCt values from RT-PCR studies comparing WT versus KO.

	Ctrl	Sham	WT 4d TBI	KO 4d TBI
NLRP3	18.982	18.836	17.645	18.739
ASC	17.186	16.774	14.953	15.652
Caspase-1	17.097	17.010	15.832	17.836
IL-1*β*	28.456	26.964	23.441	26.411

## Abbreviations

ASC: Apoptosis-associated speck-like protein containing a CARD
CCI: Controlled cortical impact
IL-1*β*: Interleukin-1*β*
KO: Knockout
NLRP3: NOD-like receptor with a pyrin domain-3
ROS: Reactive oxygen species
TBI: Traumatic brain injury
TXNIP: Thioredoxin-interacting protein
WT: Wild-type.
